# Model-informed drug development for antimicrobials: translational pharmacokinetic-pharmacodynamic modelling of apramycin to facilitate prediction of efficacious dose in complicated urinary tract infections

**DOI:** 10.1093/jac/dkae409

**Published:** 2024-11-16

**Authors:** Irene Hernández-Lozano, Vincent Aranzana-Climent, Sha Cao, Carina Matias, Jon Ulf Hansen, Edgars Liepinsh, Diarmaid Hughes, Sven N Hobbie, Carina Vingsbo Lundberg, Lena E Friberg

**Affiliations:** Department of Pharmacy, Uppsala University, Uppsala, Sweden; Department of Pharmacy, Uppsala University, Uppsala, Sweden; PHAR2, Inserm U1070, Université de Poitiers, Poitiers, France; Department of Medical Biochemistry and Microbiology, Uppsala University, Uppsala, Sweden; Department of Bacteria, Parasites & Fungi, Statens Serum Institut, Copenhagen, Denmark; Department of Bacteria, Parasites & Fungi, Statens Serum Institut, Copenhagen, Denmark; Laboratory of Pharmaceutical Pharmacology, Latvian Institute of Organic Synthesis, Riga, Latvia; Department of Medical Biochemistry and Microbiology, Uppsala University, Uppsala, Sweden; Institute of Medical Microbiology, University of Zurich, Zurich, Switzerland; Division of Clinical Bacteriology and Mycology, University Hospital Basel, Basel, Switzerland; Department of Bacteria, Parasites & Fungi, Statens Serum Institut, Copenhagen, Denmark; Department of Pharmacy, Uppsala University, Uppsala, Sweden

## Abstract

**Objectives:**

The use of mouse models of complicated urinary tract infection (cUTI) has usually been limited to a single timepoint assessment of bacterial burden. Based on longitudinal *in vitro* and *in vivo* data, we developed a pharmacokinetic-pharmacodynamic (PKPD) model to assess the efficacy of apramycin, a broad-spectrum aminoglycoside antibiotic, in mouse models of cUTI.

**Methods:**

Two *Escherichia coli* strains were studied (EN591 and ATCC 700336). Apramycin exposure–effect relationships were established with *in vitro* time–kill data at pH 6 and pH 7.4 and in mice with cUTI. Immunocompetent mice were treated with apramycin (1.5–30 mg/kg) starting 24 h post-infection. Kidney and bladder tissue were collected 6–96 h post-infection for cfu determination. A PKPD model integrating all data was developed and simulations were performed to predict bacterial burden in humans.

**Results:**

Treatment with apramycin reduced the bacterial load in kidneys and bladder tissue up to 4.3-log compared with vehicle control. *In vitro* and *in vivo* tissue time-course efficacy data were integrated into the PKPD model, showing 76%–98% reduction of bacterial net growth and 3- to 145-fold increase in apramycin potency *in vivo* compared with *in vitro.* Simulations suggested that an 11 mg/kg daily dose would be sufficient to achieve bacterial stasis in kidneys and bladder in humans.

**Conclusions:**

PKPD modelling with *in vitro* and *in vivo* PK and PD data enabled simultaneous evaluation of the different components that influence drug effect, an approach that had not yet been evaluated for antibiotics in the cUTI model and that has potential to enhance model-informed drug development of antibiotics.

## Introduction

Urinary tract infections (UTIs) are one of the most common bacterial infections,^1,2^ predominantly caused by uropathogenic *Escherichia coli* bacteria.^3,4^ These bacteria enter through the urinary meatus, ascending up the urethra into the bladder^2^ and potentially into the kidneys, which, in addition to complicating factors such as functional urinary tract abnormalities, or immune-compromised conditions, can lead to complicated UTI (cUTI) and pyelonephritis.^5^ The emergence of antibiotic resistance among Gram-negative organisms complicates UTI treatment, which highlights the need for development of new therapeutics.^2,5^

In order to predict human efficacious doses and support the development of new antibiotic therapies, a reliable prediction of human pharmacokinetics (PK) and pharmacodynamics (PD) is necessary. Traditionally, in the absence of human PK data, this has been done by extrapolating PK parameters obtained from preclinical studies.^6–8^ PKPD relationships of antibiotics are typically described by evaluating the correlation of an outcome therapeutic variable (e.g. end-of-treatment response in an animal infection model) to PK/PD indices (i.e. *f*AUC/MIC -area under the unbound concentration-time curve above MIC-, *fC*_max_/MIC -maximum unbound concentration above MIC- and *f*T > MIC -percent of time above MIC), which evaluate drug exposure in relation to the MIC of the drug.^9,10^ However, these PK/PD indices do not consider the time-course of the PK and PD of the drug, mainly due to the lack of longitudinal data generated in animal species.

The *in vitro* time-course of bacterial growth and killing together with human PK profiles have been used to develop semi-mechanistic PKPD models to predict optimal human dosages of available drugs.^6,9,11^ However, *in vitro* data are not always predictive of *in vivo* scenarios because other factors, such as immune defence mechanisms or drug distribution and metabolism, can influence the growth and death rate of bacteria in the complex environment of the infected host.^12^ Exploring not only *in vivo* drug PK but also bacterial growth dynamics in preclinical animal models *in vivo* could result in more suitable human dosages of drugs under development. Murine ascending UTI models have been previously used to assess virulence factors of uropathogenic *E. coli*, as they resemble the natural course of ascending UTI that causes pyelonephritis.^13^ In addition, these mouse models are useful tools to study antibiotic efficacy *in vivo* against UTIs with the possibility to assess bacterial burden in urine, bladder and kidneys.^14,15^

Aminoglycosides are potent broad-spectrum antibiotics proposed as highly effective treatment alternatives to overcome challenges associated with antibiotic-resistant Gram-negative bacteria.^16,17^ However, *E. coli* UTI infections have been shown to further decrease the naturally acidic pH of urine, which can compromise the antibacterial potency of aminoglycoside antibiotics.^18^ Apramycin, however, is affected to a lesser extent by clinically relevant aminoglycoside resistance mechanisms because of its unique chemical structure, different from other aminoglycosides.^19,20^

In this study, longitudinal PK and PD data from *in vitro* and *in vivo* studies were used together with PKPD modelling to assess apramycin efficacy in mouse models of cUTI. The developed PKPD model, together with a previous population PK (popPK) model developed in healthy human volunteers,^21^ was used to predict bacterial growth dynamics in humans.

## Materials and methods

### In vitro time–kill experiments

Two *E. coli* strains, EN591 (an MDR *rmtB* isolate) and ATCC 700336 (EN1085, a trimethoprim/sulfamethoxazole-resistant urinary tract infection isolate) were studied. The effect of apramycin was assessed in time–kill experiments at pH 7.4 and at pH 6 to assess possible differences in bacterial growth derived by the more acidic nature of urine compared with other tissues with neutral pH such as the blood. An overnight culture of each strain was diluted 100-fold into 2 mL prewarmed Mueller–Hinton II (MHII) broth, grown to logarithmic phase (OD_600_ = 0.1–0.3). Aliquots of 10^5^–10^6^ bacteria cfu were inoculated into propylene tubes containing the prewarmed medium with pH adjusted and buffered to ensure stability throughout the experiment.^22^ Apramycin was added to the tubes to achieve concentrations ranging from 0.25 to 8 × MIC. The tubes were incubated at 37°C and samples were taken for viable counts at 0, 1, 2, 4, 6, 8, 10, 24 and 28 h. Appropriate dilutions of each sample were made in 0.9% NaCl and spread on MHII agar plates with five glass beads (6 mm, Hecht 1401/6). After incubation at 37°C for 24 ± 4 h, cfu were counted manually. The limit of detection was 10 cfu/mL.

### Mouse PD studies

Animal experiments were approved by the local animal ethics committee (SSI, Denmark) and performed in accordance with the European Directive 2010/63/EEC and Danish licence no. 2020-15-0201-00730. An overview of the animals included in the study is given in Table S1 (available as Supplementary data at *JAC* Online). Briefly, animals were infected via transurethral inoculation with the same bacterial strains that were used for the *in vitro* experiments. Apramycin was administered subcutaneously twice a day in doses ranging from 1.5 to 30 mg/kg starting at 24 h after bacterial inoculation. Animals were sacrificed by cervical dislocation at 6, 10, 24, 30, 48, 72 and 96 h after bacterial inoculation. Kidneys and bladder tissue were harvested and homogenized in sterile 0.9% NaCl, and cfu per bladder or per two kidneys (cfu/organ) was determined after 18–22 h incubation at 35°C. Additional cfu data from previous experiments in mouse models with the same two strains were also included for modelling and simulation purposes (see Supplementary methods).

### Mouse PK studies

The PK of apramycin was evaluated in a UTI mouse model. Similar to the PD studies in mice, 6-week-old female C3H/HeJ mice received apramycin treatment 24 h after infection with the *E. coli* strain EN591. Apramycin was administered twice a day for 3 days and blood was sampled to evaluate apramycin PK at 15 min, 30 min and 1 h after the first and last dose.

### PKPD modelling

**Figure 1.**
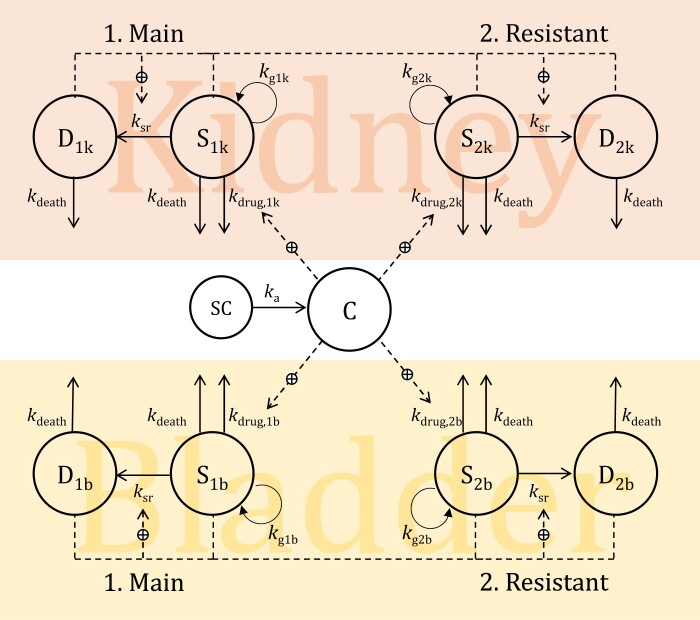
Schematic representation of the pharmacokinetic-pharmacodynamic (PKPD) model. The model includes two bacterial subpopulations, one susceptible (1. Main) and one resistant (2. Resistant). In each subpopulation, the bacteria may exist in one of two discrete states: (S) antibiotic-susceptible proliferating bacteria and (D) dormant bacteria unsusceptible to the antibiotic. The bacteria enter the D state as a response to high population densities. *k*_a_, absorption rate constant; *k_g_*, bacterial growth rate constant; *k*_death_, bacterial death rate constant; *k*_drug_, rate constant describing the drug effect (modelled as a power model); *k*_sr_, rate constant describing the transfer from the susceptible to the dormant population as a response to high population densities; k, kidney; b, bladder; SC, compartment for subcutaneous administration; C, central (plasma) compartment. This figure appears in colour in the online version of *JAC* and in black and white in the print version of *JAC*.

A PKPD model was developed by first using the *in vitro* time–kill data (Figure 1). Strain-dependent models were developed, considering the different pH under which the experiments were performed. The structural model consisted of two bacterial subpopulations: 1: a main population susceptible to apramycin, and 2: a subpopulation with decreased susceptibility (Figure 1). Both subpopulations consisted of two states, a growing drug-susceptible state (S) and a dormant unsusceptible state (D), which the bacteria enter as a response to high population densities. In addition, a model evaluating an adaptive resistance mechanism for the bacteria was also tested. The drug acted by increasing the death rate in the S state by a rate *k*_drug_ and was modelled by a power model. An *E*_max_ model was tested to assess the concentration–effect relationship. The natural death rate of bacteria *in vitro* (*k*_d_) was set to a fixed value of 0.179 h^−1^ based on previously published data.^23^ A rate constant (*k*_ada_), describing the drug-driven transfer from the susceptible to the resistant population, was included in the model rather than assuming initial pre-existing percentages of each subpopulation.^24^ The *in vivo* efficacy data together with the *in vitro* data were then added to the best-fitting model for analysis and re-estimation of the PD parameters together with a previously developed PK model for apramycin in healthy and infected mice using an unbound fraction of apramycin in plasma of 91.6%.^25,26^ Since the mice were immunocompetent, the value of *k*_d_ was initially not fixed for the *in vivo* modelling in order to consider factors, such as host defence mechanisms, influencing bacterial death in the living organism.^12^ For animals infected with the bacterial strain ATCC 700336, *k*_d_ had to be fixed to the *in vitro* literature value of 0.179 h^−1^ as the scarcity and variability of the observed data did not allow estimation of physiologically reasonable values.

The final PKPD model was then used to predict bacterial killing from a different study in mice infected with the EN591 bacterial strain for which only two timepoints (at 24 and 96 h after bacterial inoculation—at start and end of treatment) were available and which had been treated with lower doses (0.03, 0.1, 0.3 and 1 mg/kg twice daily) of apramycin; as well as in a different UTI mouse model (infected with ATCC 700336 4 days before start of treatment) treated with apramycin at doses ranging from 0.05 to 51.2 mg/kg twice daily (see Table S1). Predictions were made without allowing for re-estimation of parameters and using the covariance matrix from a sampling–importance resampling (SIR) analysis to include parameter uncertainty in the simulations.

The developed PKPD model assumed that the apramycin central unbound concentration corresponded to the site-of-action concentration. This assumption was validated by the development of a physiologically based PK (PBPK) model that was used to predict the apramycin in plasma and kidney interstitium of mice. The model was developed based on the physicochemical properties of apramycin and the physiology of a 20 g average-weight mouse.

### Prediction of human efficacy

A previously developed human popPK for apramycin^21^ (Figure S1) combined with the PD component of the mouse PKPD model was used to simulate bacterial burden and antibacterial effect of apramycin in humans over 48 h. An unbound fraction of 92.9% in human plasma was applied.^26^ Unbound plasma concentrations of apramycin were used to drive an antibacterial effect in kidneys and bladder. Predictions were performed for single doses of 0.3, 1.2, 3.6, 10.8 and 30 mg/kg administered as an IV infusion over 30 min, in a 75 kg adult with creatinine clearance of 120 mL/min. The doses selected for the predictions were those used in the Phase I trial,^21^ where a 30 mg/kg dose was found to have an acceptable tolerability in the study participants. Based on the mouse data, initial bacterial densities were set to 10^6^ and 10^5^ per organ for kidneys and bladder, respectively, and pH was assumed to be 7.4 and 6 for the kidneys and bladder, respectively.

### Data analysis and software

Model development, visual predictive checks (VPCs) and SIR^27^ were performed using the nonlinear mixed-effects modelling software NONMEM v7.5.0 (ICON Development Solutions, San Antonio, TX, USA) with Perl-speaks-NONMEM v3.5.0. Model selection was based on the difference in objective function value (dOFV) where a change of ≥3.84 was considered as a statistical difference at the 5% significance level (*P *< 0.05). In addition to dOFV, model development was guided based on parameter precision and goodness-of-fit, including the simulation-based VPCs. Parameter sharing between strains was done for simplification of the model if supported by dOFV and parameter precision. For residual unexplained variability, additive error models were used. R v4.2.0 (R Foundation for Statistical Computing, Vienna, Austria),^28^ with the Xpose and mrgsolve packages, was used for data management, graphical evaluation and simulations. In addition, the modelling software PK-sim^®^ v11.1 (Open Systems Pharmacology Suite, Bayer Technology Services, Leverkusen, Germany) was used for simulations.

## Results

### In vitro time–kill curves and PD modelling

**Figure 2.**
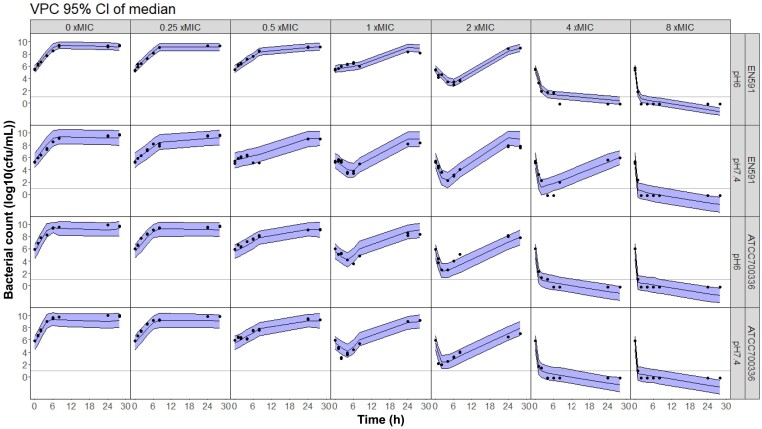
Visual predictive checks (VPCs) of the final model based on pH-adjusted *in vitro* time–kill data. Dots represent observed data, lines represent the 95% CI of the median (shaded area). Each panel represents data for a specific bacterial strain (namely, EN591 and ATCC 700336) under different pH conditions (pH 6 or pH 7.4) at different apramycin concentrations. Data below the limit of quantification (10 log_10_ cfu/mL) are plotted as −0.2 (MIC for EN591: 8 and 32 mg/L at pH 7.4 and pH 6, respectively; MIC for ATCC 700336: 4 and 16 mg/L at pH 7.4 and pH 6, respectively). This figure appears in colour in the online version of *JAC* and in black and white in the print version of *JAC*.

The general structure of the PKPD model is depicted in Figure 1, and parameter estimates and the 95% CIs are summarized in Table 1. The growth rate constant was 28% lower in the EN591 strain compared with the ATCC 700336 strain. The model could well capture the killing and regrowth for both strains, and VPCs demonstrated a good fit to the time–kill data (Figure 2). Drug effect was normalized to MIC, which was four times higher at pH 6 (MIC_EN591_ = 32 mg/L, MIC_ATCC700336 _= 16 mg/L) compared with pH 7.4 (MIC_EN591_ = 8 mg/L, MIC_ATCC700336_ = 4 mg/L) for both strains. This allowed using the same parameters to describe drug effect at both pH levels for the strain ATCC 700336 without significantly changing the model fit. For EN591, differences in bacterial regrowth at 4 × MIC between pH 6 and pH 7.4 conditions resulted in different drug effect parameters.

**Table 1.** Parameter estimates and 95% CIs determined by sampling–importance resampling for the final models

Parameter	Unit	Description	Strain	Value	95% CI
***In vitro* PD parameters**					
*k*_g_	h^−1^	Bacteria growth rate constant susceptible population	EN591	1.64	1.54–1.77
			ATCC700336	2.27	2.05–2.59
Reduk_g_	%	Fractional reduction in growth rate for the resistant subpopulation	Both	0.63	0.05–5.51
*k*_d_	h^−1^	Natural bacterial death rate constant	Both	0.179	Fixed
*B*_max_	log_10_ cfu/mL	Maximum bacterial density	Both	9.18	8.97–9.38
Inoc	log_10_ cfu/mL	Initial bacterial density	Both	5.53	5.44–5.64
γ	—	Power on drug effect	Both	1	Fixed
SlopeS	h^−1^	Rate constant for apramycin effect normalized by MIC in the susceptible subpopulation	EN591 pH 6	1.31	1.18–1.45
			EN591 pH 7	2.37	1.89–2.85
			ATCC700336	2.99	2.38.3.80
SlopeR	h^−1^	Rate constant for apramycin effect normalized by MIC in the resistant subpopulation	EN591 pH 6	0.385	0.353–0.420
			EN591 pH 7	0.254	0.232–0.280
			ATCC700336	0.776	0.604–0.967
*k*_ada_ × 1000	h^−1^	Rate constant for drug-driven transfer from susceptible to resistant	EN591 pH 6	0.080	0.033–0.153
			EN591 pH 7	0.028	0.012–0.063
			ATCC700336	0.210	0.013–0.581
***In vivo* PD parameters**					
*k*_g1k_	h^−1^	Bacteria growth rate constant susceptible population in kidneys	EN591	0.694	0.432–1.064
			ATCC700336	0.205	0.187–0.228
Reduck_gk_	%	Fractional reduction in growth rate for the resistant subpopulation in kidney	EN591	25.4	16.8–34.5
			ATCC700336	6.9	0.5–16.9
*k*_d,k_	h^−1^	Bacterial death rate constant in kidney	EN591	0.526	0.348–0.745
			ATCC700336	0.179	Fixed
SlopeS_k_	h^−1^	Rate constant for apramycin effect normalized by MIC in the susceptible subpopulation in kidney	Both	9.05	5.41–13.11
SlopeR_k_	h^−1^	Rate constant for apramycin effect normalized by MIC in the resistant subpopulation in kidney	Both	0.066	0.026–0.109
*k*_ada,k_	h^−1^	Rate constant for drug-driven transfer from susceptible to resistant in kidney	Both	0.031	0.006–0.062
*B*_max,k_	log_10_ cfu/organ	Maximum bacterial density in kidneys	Both	6.49	Fixed
Inoc_k_	log_10_ cfu/organ	Initial bacterial density in kidneys	Both	5.70	5.38–6.04
*k*_g1b_	h^−1^	Bacteria growth rate constant susceptible population in bladder	EN591	1.51	1.09–1.84
			ATCC700336	0.228	0.206–0.254
Reduck_gb_	%	Fractional reduction in growth rate for the resistant subpopulation in bladder	EN591	25.2	19.2–30.3
			ATCC700336	6.9	0.5–16.9
*k*_d,b_	h^−1^	Bacterial death rate constant in bladder	EN591	1.16	0.85–1.39
			ATCC700336	0.179	Fixed
SlopeS_b_	h^−1^	Rate constant for apramycin effect normalized by MIC in the susceptible subpopulation in bladder	Both	191	58–1147
SlopeR_b_	h^−1^	Rate constant for apramycin effect normalized by MIC in the resistant subpopulation in bladder	Both	0.276	0.153–0.403
*k*_ada,b_	h^−1^	Rate constant for drug-driven transfer from susceptible to resistant in bladder	Both	1.35	0.43–8.86
*B*_max,b_	log_10_ cfu/organ	Maximum bacterial density in bladder	Both	7.07	Fixed
Inoc_b_	log_10_ cfu/organ	Initial bacterial density in bladder	Both	4.42	3.77–4.96
γ	—	Power on drug effect (kidneys and bladder)	Both	1	Fixed

### Mouse PKPD experiments and PKPD modelling

The plasma concentration profiles of mice infected with the bacterial strain EN591 after the first and last dose of apramycin were well described by a previously developed one-compartment model^25^ with a dose-dependent *k*_a_ (Figure S2).

Bacterial burden in kidneys and bladder (cfu/organ) at 96 h after bacterial inoculation (i.e. 72 h after start of treatment) was reduced by at least 2-log in comparison with the start of treatment (24 h after inoculation) and with respect to vehicle control. Similarly, reduction of bacterial burden at 72 h after start of treatment for animals infected for 4 days with the ATCC 700336 strain was also at least 2-log for doses of 3.2, 12.8 and 51.2 mg/kg with respect to vehicle control (Figure S3).

**Figure 3.**
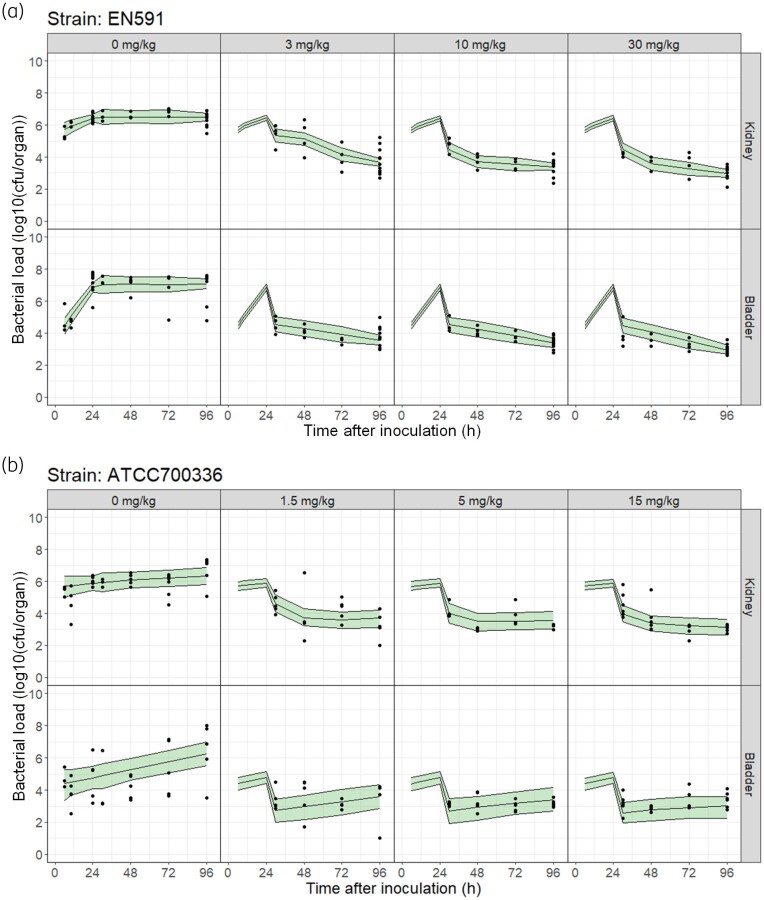
Visual predictive checks of the final *in vivo* PKPD model. The model was developed from combined plasma PK and kidney and bladder PD data in mice infected with either (a) EN591 or (b) ATCC 700336 bacterial strains. Dots represent observed PD data (log_10_ cfu/organ), and lines represent the 95% CI of the median (shaded area). Each panel represents data for an organ (either kidney or bladder) at different apramycin doses. Apramycin was administered subcutaneously twice daily for 3 days starting at 24 h after bacterial inoculation. This figure appears in colour in the online version of *JAC* and in black and white in the print version of *JAC*.

The final model resulted in a good fit to the data (Figure 3). Parameter uncertainty was low for all parameters, except the drug effect parameters in bladder (Table 1), which showed wide CIs and high relative standard error (%RSE) in the parameter estimates (SlopeS_b_: 159%, *k*_ada,b_: 178%). For both strains, the net growth rate constant (*k*_net_ = *k*_g_ − *k*_d_) was lower *in vivo* than *in vitro* (EN591: −91% and −76% for *k*_net,kidney_ and *k*_net,bladder_, respectively; ATCC 700336: −99% and −98% for *k*_net,kidney_ and *k*_net,bladder_, respectively). In addition, the apramycin effect in the susceptible population (SlopeS), as well as *k*_ada_ (rate constant describing the transfer from susceptible to resistant populations), were notably higher *in vivo* than *in vitro*. The initial bacterial burden at 6 h after inoculation was estimated to be 5.70 and 4.42 (log_10_cfu/organ) for kidney and bladder, respectively, which agrees with the observed values. Drug effect parameters in both the kidneys and bladder were shared for both cUTI mouse models without a significant impact on model fit.

**Figure 4.**
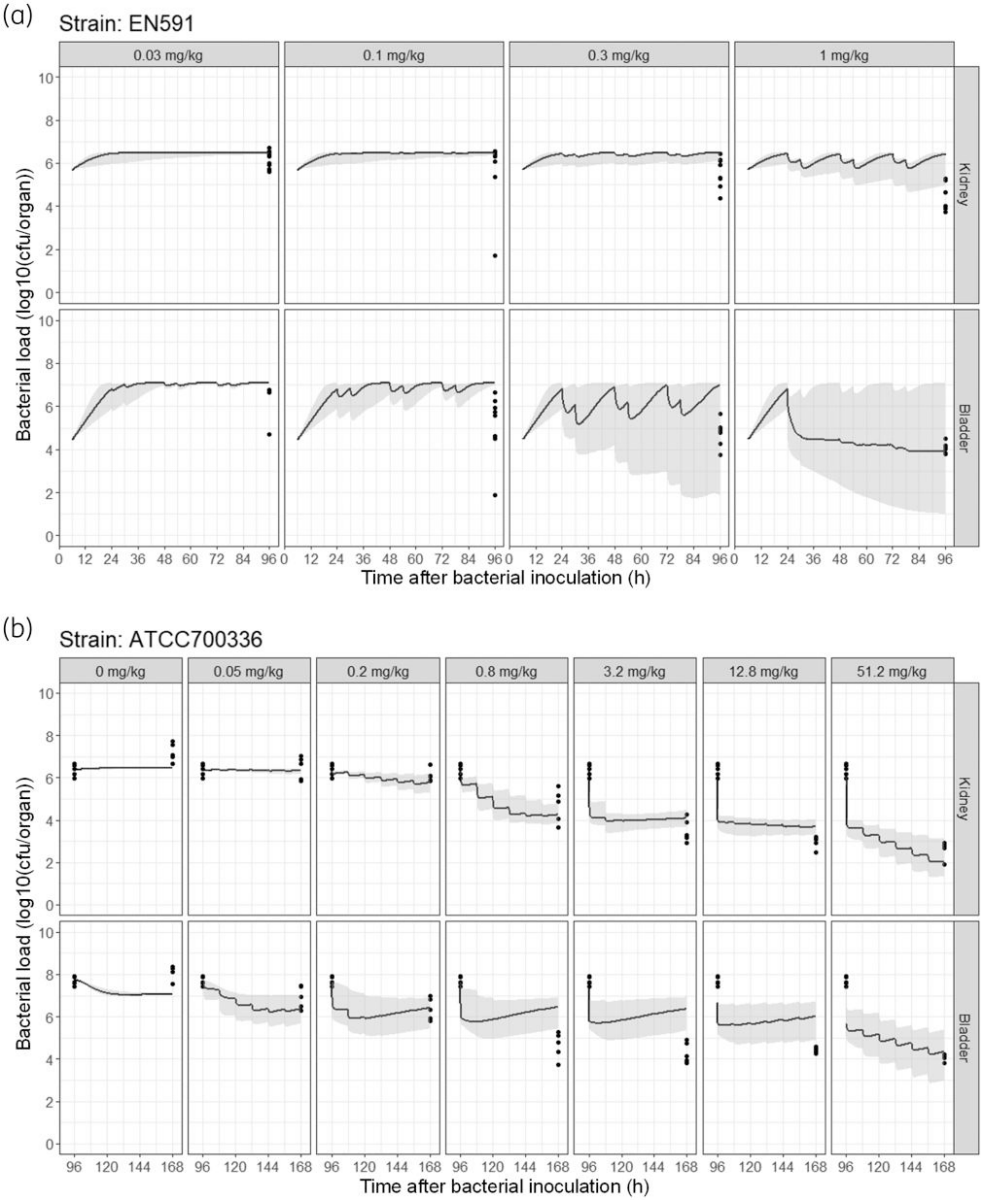
*In vivo* PD predictions in mice. Predictions in mice infected with either (a) EN591 or (b) ATCC 700336 bacterial strains that underwent different dosing regimens of apramycin and were not included in the original modelling. Dots represent observed PD data (log_10_ cfu/organ), and lines represent the 95% CI of the median (shaded area) from 500 simulations considering RSEs in all model parameters. Each panel represents data for an organ (either kidney or bladder) at different apramycin doses. Apramycin was administered subcutaneously twice daily for 3 days starting at either (a) 24 h or (b) 96 h after bacterial inoculation.

Simulations with the developed PKPD model in the same EN591 cUTI mouse model that underwent treatment with lower apramycin doses slightly underpredicted bacterial burden for the apramycin doses 0.3 and 1 mg/kg in the kidney (Figure 4a). In addition, simulations in mice infected with the ATCC 700336 bacterial strain for 4 days before start of treatment predicted the bacterial killing well, except for the 0.8, 3.2 and 12.8 mg/kg apramycin doses, for which the bacterial burden in bladder was slightly underpredicted (Figure 4b).

The PBPK model showed the expected PK profile of apramycin in the kidney interstitium after subcutaneous administration of apramycin to be comparable to that of plasma (Figure S4a). The PBPK model was also used to simulate apramycin PK in humans after a single IV infusion, which showed that PK of apramycin in plasma and kidney interstitium were comparable (Figure S4b) and further justified the use of unbound apramycin plasma concentration as the site-of action concentrations for modelling purposes.

### Prediction of human efﬁcacy

**Figure 5.**
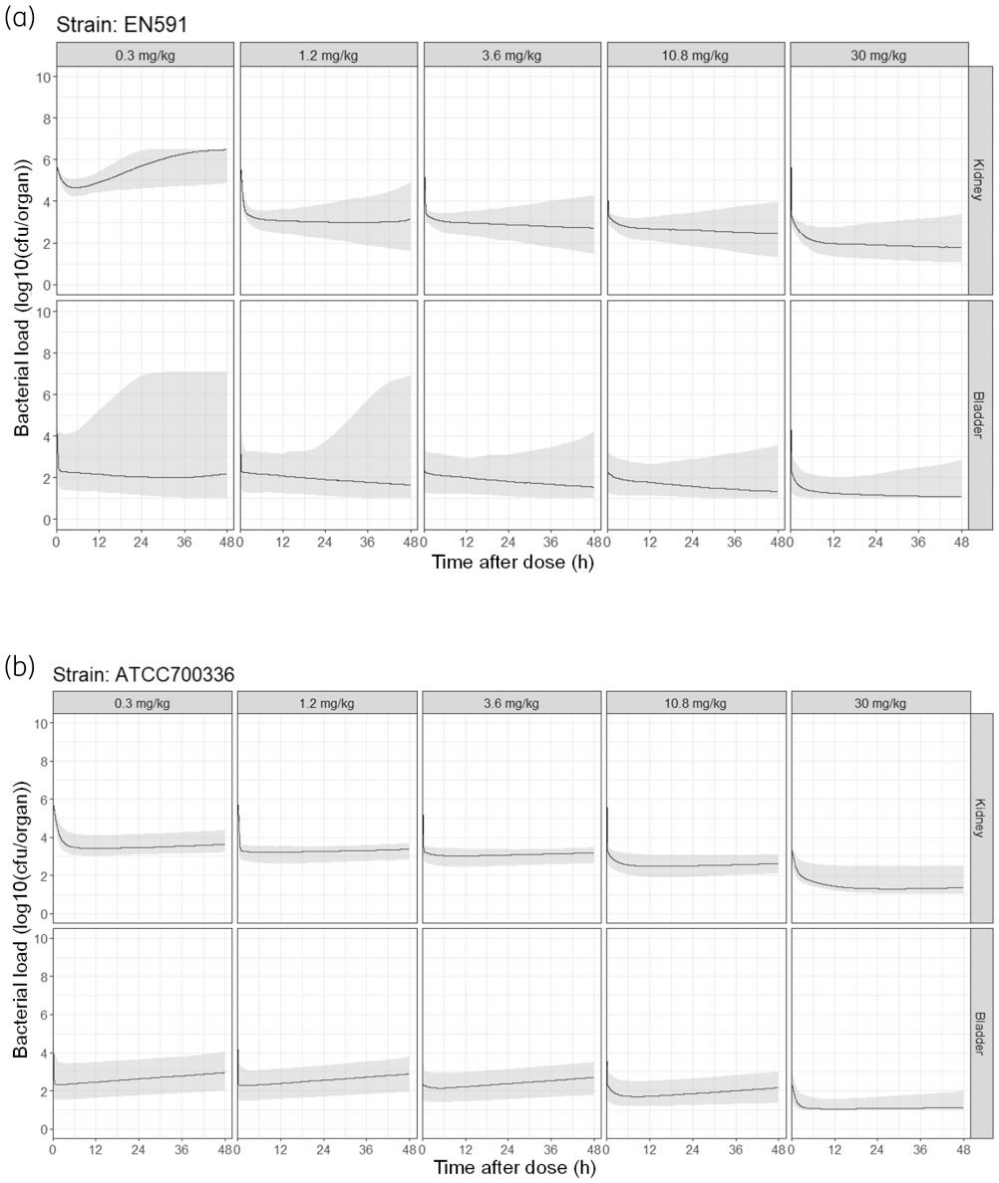
Predicted efficacy in humans for the studied strains. (a) EN591 and (b) ATCC 700336. Expected bacterial densities (log_10_ cfu/organ) are depicted for 48 h after a 30 min IV infusion of apramycin at 0.3, 1.2, 3.6, 10.8 or 30 mg/kg. Human unbound plasma PK profiles were predicted for a 75 kg patient with a creatinine clearance of 120 mL/min. Each panel represents data for an organ (either kidney or bladder) at the different apramycin doses explored previously in human healthy volunteers. Lines represent median and shaded area represents 95% CI of simulations considering inter-individual variability (IIV), residual error and RSEs in all model parameters.

The predicted bacterial burden over time in human kidneys and bladder after 0.3–30 mg/kg single IV doses of apramycin is shown in Figure 5. A single dose of 10.8 mg/kg was predicted to be sufficient to result in stasis for both strains.

## Discussion

In this study, we evaluated apramycin efficacy in two mouse models of cUTI by using PKPD modelling together with *in vitro* static PD data and *in vivo* preclinical dynamic PK and PD data. Although mouse models of ascending cUTI have been previously used to assess the *in vivo* effect of different antibiotics,^14,29^ and few of these models have focused on assessing the reduction of bacterial burden at the end of treatment with PK/PD indices,^15,30,31^ dynamic PKPD relationships have to our knowledge not yet been evaluated in the cUTI model. The strength of this study is the availability of longitudinal *in vivo* control bacterial burden data at 6, 10, 24, 30, 48, 72, 96 and 168 h after bacterial inoculation (Figure S3), which provides better insights into bacterial growth and death *in vivo* than the commonly performed studies using the PK/PD index methodology,^9^ in which only two timepoints are available from *in vivo* data. Thereby, translational aspects, such as time-dependence and species-differences in PK as well as the different components that influence drug effect may be better accounted for.

The time intervals between inoculation and the start of treatment, as well as the time of bacterial assessment after the start of treatment, have not been standardized for UTI mouse models, and have ranged from 24 to 96 h for the first bacterial assessment after inoculation, which does not allow assessment of bacterial growth and antibiotic killing *in vivo* early after inoculation. This is opposite to other mouse infection models, such as the lung or the thigh mouse infection models, in which bacterial burden is commonly assessed at the site of infection 1–2 h after inoculation (start of treatment for efficacy studies) and 24 h after start of treatment.^25,26^ In these models, the PKPD of apramycin was previously defined with a similar modelling methodology to the one presented in this work.^25,26^ In addition, a proof-of-concept study showed a similar potency of apramycin against uropathogenic bacterial isolates but with a significantly lower nephrotoxicity compared with other aminoglycoside antibiotics.^22^ Moreover, the lack of availability of dynamic data also after start of treatment in previous studies with the mouse models of thigh and lung infection^25,26^ poses a challenge to estimate PD parameters describing drug effect. The current study extends these previous results by quantifying the dynamic *in vivo* PD data at 6, 24, 48 and 72 h after start of treatment with apramycin (Figure S3), supported by pH-adjusted time–kill experiments *in vitro*, allowing evaluation of model differences between *in vitro* and *in vivo* data, not only regarding bacterial growth but also drug effect.

Although the apramycin antibacterial effect *in vivo* was the same for both strains, the net bacterial growth (*k*_net_) of ATCC 700336 was 80% and 86% lower in the kidney and bladder, respectively, compared with EN591. Interestingly, this was the opposite for the bacterial growth *in vitro*, where a 43% higher net growth was noted for ATCC 700336 compared with EN591. However, as these mouse models were immune-competent, bacterial growth and death *in vivo* were likely influenced by the host immune system, which was also reflected by the significant reduction (up to 91% for EN591 and up to 99% for ATCC 700336) of bacterial net growth *in vivo* compared with *in vitro.* This significant reduction in bacterial net growth *in vivo* compared with *in vitro* could result from the optimal conditions of the *in vitro* environment that stimulate bacterial growth, such as the lack of external mechanisms or the addition of nutrients to the *in vitro* growth media.

The apramycin doses given to the animals infected with the bacterial strain ATCC 700336 were half those given to animals infected with EN591 based on the lower *in vitro* MIC of apramycin. Nevertheless, for both strains in mice, a dose of at least 10 mg/kg twice daily (i.e. 20 mg/kg daily) was enough to achieve bacterial stasis (Figure 3). This translated into a single dose of 10.8 mg/kg to be efficient in humans (Figure 5). Similarly, previous preclinical *in vitro* and *in vivo* studies with apramycin concluded that a daily dose of 30 mg/kg in humans is a promising and well-tolerated^21^ dosing regimen for pneumonia and UTI^25,32^ using AUC/MIC as the PK/PD index to be predictive of bacterial load reduction, based on mouse thigh and lung infection models.^25,26^ The effective doses found for apramycin were two to four times those for other aminoglycosides such as gentamicin or amikacin, respectively, which agrees with the drugs’ differences in MIC at different pHs for susceptible strains.

It is important to consider that longitudinal PK profiles in the kidney were not available and therefore the unbound apramycin concentration in the central compartment was assumed to drive the apramycin effect at the site of action based on the similar PK profiles in plasma and kidney interstitium simulated from the PBPK model. Although caution is needed when using PBPK models to predict tissue concentrations,^33^ it is not always the case that PBPK models do not predict interstitial fluid concentrations. The concentration in the central compartment was normalized to the *in vitro* MIC assuming pH 7.4 and pH 6 for the kidneys and bladder, respectively, in order to take into account bacterial growth differences that might be derived from pH differences in the tissues.^22^ Time–kill experiments reported in previous studies have typically not been designed to reflect the acidic urinary environment, although the bactericidal activity of antibiotics may change significantly with changes in pH.^22,34^ In this study, a pH-buffered growth medium was used in order to ensure that the pH remained stable throughout the *in vitro* experiments.

Although the use of longitudinal data for PKPD modelling proved useful in predicting the effect of apramycin, it should be highlighted that a significant number of animals were required to obtain time-courses of bacterial burden. This study supports the use of a PKPD model developed from *in vitro* data, in combination with information on *in vivo* bacterial growth, to minimize the number of required animals by study design.

Altogether, the present study illustrates how the combination of dynamic PK and PD data with PKPD modelling can be used to make predictions of drug exposure and effect in patients. This approach enhances the significance of preclinical *in vitro* and *in vivo* data allowing exploration of different scenarios. The inclusion of longitudinal PD data allows simultaneous assessment, not only of bacterial burden but also of the different components influencing drug effects in the cUTI mouse models. Together with previous studies, the analysis suggests a promising efficacy of apramycin and supports further studies in patients, with the expectation that an 11 mg/kg single dose is efficacious against Gram-negative UTIs. In conclusion, the present study strengthens the potential of model-informed drug development in the translation of new antibiotics for clinical therapy.

## Supplementary Material

dkae409_Supplementary_Data

## Supplementary data

Supplementary methods, Table S1 and Figures S1 to S4 are available as Supplementary data at *JAC* Online.
