# Supplementary material for: Model-informed drug development for antimicrobials: translational pharmacokinetic-pharmacodynamic modelling of apramycin to facilitate prediction of efficacious dose in complicated urinary tract infections
Source: J Antimicrob Chemother. 2024 Nov 16;80(1):301–10. doi: 10.1093/jac/dkae409 (PMC11695905; doi:10.1093/jac/dkae409)
Supplement: dkae409_Supplementary_Data [file dkae409_supplementary_data.docx]

**SUPPLEMENTARY MATERIAL**

**Model-informed drug development for antimicrobials: translational pharmacokinetic-pharmacodynamic modelling of apramycin to facilitate prediction of efficacious dose in complicated urinary tract infections**

Irene Hernández-Lozano, Vincent Aranzana-Climent, Sha Cao, Carina Matias, Jon Ulf Hansen, Edgars Liepinsh, Diarmaid Hughes, Sven N. Hobbie, Carina Vingsbo Lundberg, Lena E. Friberg

**Supplementary methods**

*Mouse PD experiments*

6-week-old female C3H/HeJ mice (mean weight 16.8 g, range 12.3-21.2 g), obtained from The Jackson Laboratory (US), were housed at a SPF facility at Statens Serum Institut, Denmark. The mice were randomized according to in-house protocol into Type 3 macrolone cages with bedding from Tapvei and Tapvei S-brick Aspen and Diamond twists enrichment. Further, the animals were offered Enviro-Dri nesting material and cardboard houses (Bio-serv) and kept under controlled environmental conditions 22 ± 2°C, 55 ± 10 % humidity, 8-10 air changes per hour, 12 h light/dark cycle, with free access to water and standard laboratory rodent diet (Teklad Global diet 2916C-Envigo). Human contact was during hours with light. Three days before the study start and during the study the mice had free access to 5 % glucose in the drinking water to induce diuresis. Approximately 60 minutes before bacterial inoculation, mice were treated orally with 45 µL nurofen (20 mg ibuprofen/mL corresponding to 30 mg/kg) as pain relief and urine was removed from the bladder by gently pressing the abdomen. Mice were anaesthetized (Zolezepam 15 mg/kg; Tiletamin 15 mg/kg; Narcozyl 24 mg/kg; Torbugesic: 0.3 mg/kg, subcutaneous injection in the scruff of the neck) and a syringe with a catheter, containing the bacterial suspension, was inserted via the urethra into the bladder and the inoculum (5×10^7^ CFU) was slowly injected. The same two strains of *E. coli* were used as for the *in vitro* experiments. After inoculation, mice were kept in their cages at 35°C under surveillance (3-4 hours) until fully awake and then transferred to racks at room temperature. During the experiment, mice were monitored at least twice daily to assess a clinical score. Apramycin stock solution at 150 mg/mL was further diluted in sterile saline solution to reach the used concentrations corresponding to 1.5, 5 and 15 mg/kg for mice infected with ATCC 700336 strain and 3, 10 and 30 mg/kg injected into mice infected with EN591 strain. Mice were treated BID, subcutaneously in the scruff of the neck or flank, with 0.2 mL of test compound or vehicle at 24, 30, 48, 54, 72 and 78 hours after bacterial inoculation. None of the mice met the humane endpoint during the experiment or showed adverse reactions to treatment. Animals were sacrificed by cervical dislocation at 6, 10, 24, 30, 48, 72 and 96 hours after bacterial inoculation. Bladder and kidneys were aseptically removed from the animals and stored at -80°C until homogenised in 0.5 and 1 mL of saline solution, respectively. The primary experimental outcome was CFU quantified from homogenates of both kidneys (CFU/kidneys) or bladder (CFU/bladder), serially diluted, plated on 5% horse blood 9 cm agar plates and counted after 18-22 hour incubation at 35˚C.

Additional C3H/HeJ female mice infected with the EN591 strain (5×10^7^ CFU) were treated with 0.03, 0.1, 0.3, 1, 3, 10 and 30 mg/kg apramycin BID, subcutaneously at 24, 30, 48, 54, 72 and 78 hours after bacterial inoculation. CFU in the kidneys and bladder were determined at 96 hours after bacterial inoculation (i.e. 72 hours after start of treatment).

Additionally, previously obtained data from a different cUTI mouse model were used. Female C3H/HeJ mice (7 weeks of age) were provided by the Jackson Laboratory, USA. Animals were acclimated for one week prior to use maintained in a controlled environment (temperature: 20-24°C, humidity: 30-70%, and 12 hours light/dark cycle) at the Pharmacology Discovery Services Taiwan, Ltd. Free access to sterilized standard lab diet [MFG (Oriental Yeast Co., Ltd., Japan)] and autoclaved tap water (or water with 5% glucose) were granted for the study period. Animals were inoculated with an *E. coli* suspension (ATCC 700336) by transurethral injection into the bladder using a urinary catheter. The target inoculum was 10^9^ CFU/ mouse. Apramycin was subcutaneously administered at 0.05, 0.2, 0.8, 3.2, 12.8, and 51.2 mg/kg BID (Q12h) for three consecutive days starting at 96 hours after bacterial inoculation. One no-treatment control group was sacrificed at 96 hours after infection to determine the initial bacterial counts in the kidneys and bladder at the time of first dose administration. Mice treated with apramycin were sacrificed 168 hours after infection (i.e. 72 hours after start of treatment) and CFU/organ were determined.

**Table S1.** Summary of animals included in the study.

|  |  | Sampling time after bacterial inoculation (hours) | | | | | | | |  |
| --- | --- | --- | --- | --- | --- | --- | --- | --- | --- | --- |
|  |  | 6 | 10 | 24 | 30 | 48 | 72 | 96 | 168 |  |
| Strain and study number | Dose (mg/kg)  b.i.d. | Number of animals | | | | | | | | Total |
| EN591  Study 1  Study 2 | Control^a^ | 4 | 4 | 4+8 | 4 | 4 | 4 | 4+7 |  | 43 |
|  | 30^a^ |  |  |  | 4 | 4 | 4 | 4+8 |  | 24 |
|  | 10^a^ |  |  |  | 4 | 4 | 4 | 4+8 |  | 24 |
|  | 3^a^ |  |  |  | 4 | 4 | 4 | 4+8 |  | 24 |
| EN591  Study 2 | 1 |  |  |  |  |  |  | 8 |  | 8 |
|  | 0.3 |  |  |  |  |  |  | 8 |  | 8 |
|  | 0.1 |  |  |  |  |  |  | 8 |  | 8 |
|  | 0.03 |  |  |  |  |  |  | 8 |  | 8 |
| ATCC 700336  Study 1 | Control | 4 | 5 | 6 | 5 | 5 | 6 | 5 |  | 36 |
|  | 15 |  |  |  | 6 | 6 | 6 | 6 |  | 24 |
|  | 5 |  |  |  | 4 | 5 | 5 | 5 |  | 19 |
|  | 1.5 |  |  |  | 5 | 5 | 5 | 5 |  | 20 |
| ATCC 700336  Study 3^b^ | Control |  |  |  |  |  |  | 5 | 5 | 10 |
|  | 51.2 |  |  |  |  |  |  |  | 5 | 5 |
|  | 12.8 |  |  |  |  |  |  |  | 5 | 5 |
|  | 3.2 |  |  |  |  |  |  |  | 5 | 5 |
|  | 0.8 |  |  |  |  |  |  |  | 5 | 5 |
|  | 0.2 |  |  |  |  |  |  |  | 5 | 5 |
|  | 0.05 |  |  |  |  |  |  |  | 5 | 5 |
|  |  |  |  |  |  |  |  |  |  | 286 |

^a^ The addition of two digits refers to the inclusion of animals from Study 1 and Study 2, respectively

^b^ Animals in Study 3 were infected 96 hours prior to start of treatment, while animals in Studies 1 and 2 were infected only 24 hours prior to start of treatment.


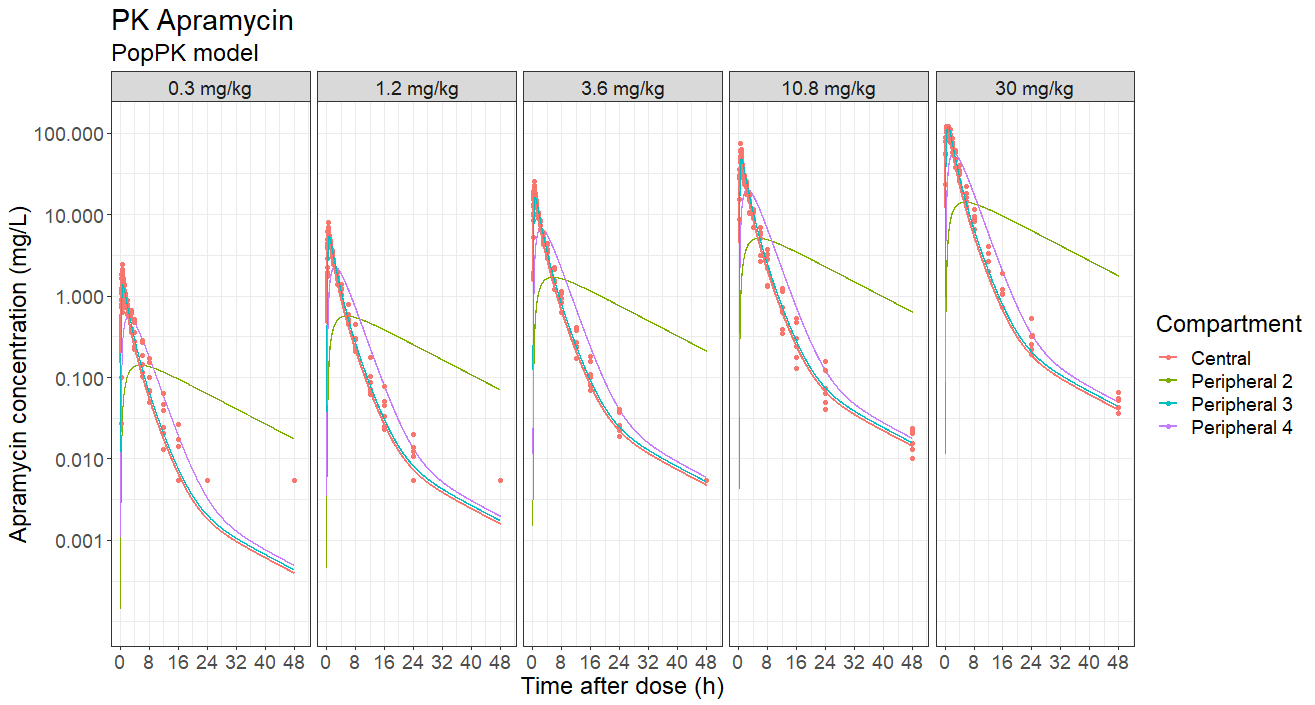


**Figure S1.** *In vivo* PK data in plasma of healthy humans. Dots represent observed data after single intravenous 30 min infusion of apramycin and lines represent predictions with the previously developed popPK model for apramycin ^2^.


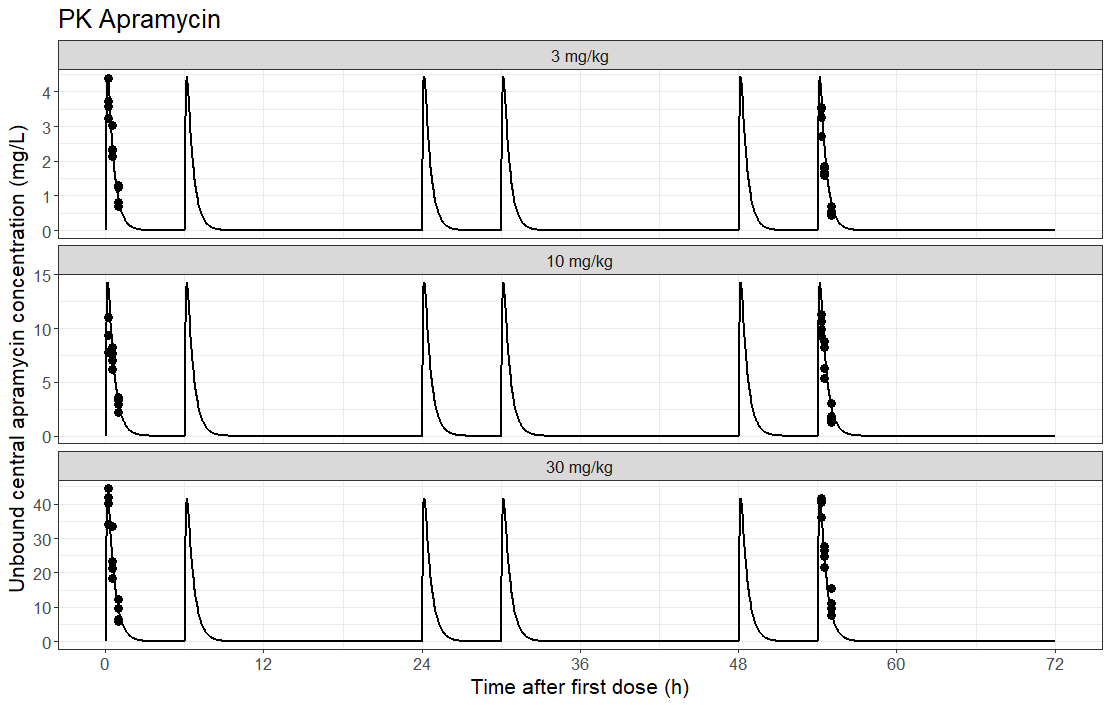


**Figure S2.** *In vivo* PK data in plasma of mice infected with the EN591 bacterial strain. Dots represent observed data after first and last dose of apramycin (subcutaneously administered twice daily for three days) and lines represent predictions with the previously developed preclinical PK model for apramycin ^1^.


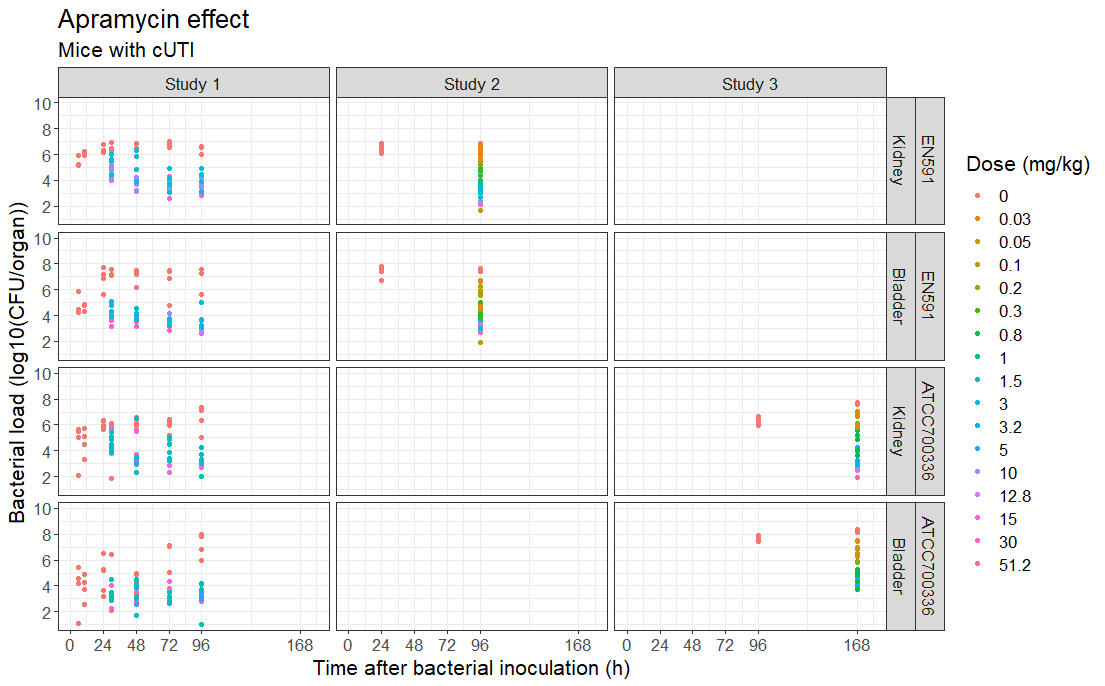


**Figure S3.** *In vivo* pharmacodynamic data (log_10_(CFU/organ)) in kidneys and bladder of mice infected with either EN591 or ATCC 700336 *E. coli* strains. Different colours represent different dosing regimens of apramycin (mg/kg b.i.d.). cUTI: complicated urinary tract infection.


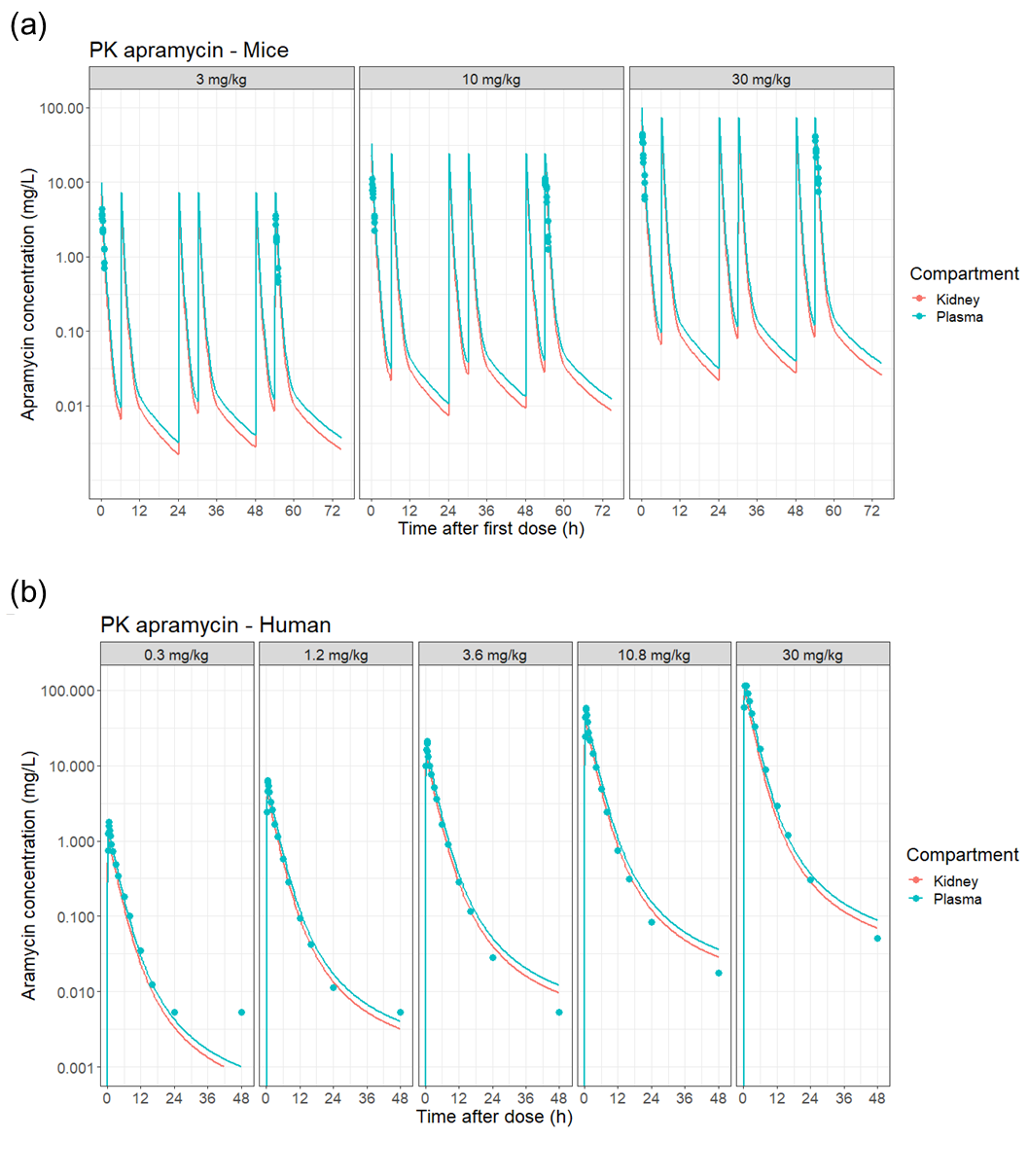


**Figure S4.** *In vivo* PK data in plasma and kidney of (a) infected mice with the EN591 bacterial strain and (b) human healthy volunteers. Dots represent observed data in plasma and lines represent predictions in plasma (blue) and kidney interstitium (red). In mice, apramycin was subcutaneously administered twice daily for three days starting 24 hours after bacterial infection. In humans, a single apramycin dose was administered intravenously as a continuous infusion for 30 minutes.

**References**

1. Sou T, Hansen J, Liepinsh E, *et al.* Model‐Informed Drug Development for Antimicrobials: Translational PK and PK/PD Modeling to Predict an Efficacious Human Dose for Apramycin. *Clin Pharma and Therapeutics* 2021; **109**: 1063–73.

2. Zhao C, Chirkova A, Rosenborg S, *et al.* Population pharmacokinetics of apramycin from first-in-human plasma and urine data to support prediction of efficacious dose. *Journal of Antimicrobial Chemotherapy* 2022; **77**: 2718–28.
